# A Semi Rigid Novel Hydroxamate AMPED-Based Ligand for ^89^Zr PET Imaging

**DOI:** 10.3390/molecules26195819

**Published:** 2021-09-25

**Authors:** Lisa Russelli, Francesco De Rose, Loredana Leone, Sybille Reder, Markus Schwaiger, Calogero D’Alessandria, Lorenzo Tei

**Affiliations:** 1 Department of Nuclear Medicine, Klinikum Rechts der Isar TU München, Ismaningerstraße 22, 81675 Munich, Germany; lisa.russelli@tum.de (L.R.); francesco.de-rose@tum.de (F.D.R.); sybille.reder@mri.tum.de (S.R.); markus.schwaiger@tum.de (M.S.); 2 Department of Science and Technological Innovation, Università del Piemonte Orientale, Viale T. Michel 11, 15121 Alessandria, Italy; loredana.leone@uniupo.it

**Keywords:** zirconium-89, polydentate chelators, hydroxamates, PET-imaging, labeling

## Abstract

In this work, we designed, developed, characterized, and investigated a new chelator and its bifunctional derivative for ^89^Zr labeling and PET-imaging. In a preliminary study, we synthesized two hexadentate chelators named AAZTHAS and AAZTHAG, based on the seven-membered heterocycle AMPED (6-amino-6-methylperhydro-1,4-diazepine) with the aim to increase the rigidity of the ^89^Zr complex by using *N*-methyl-*N*-(hydroxy)succinamide or *N*-methyl-*N*-(hydroxy)glutaramide pendant arms attached to the cyclic structure. *N*-methylhydroxamate groups are the donor groups chosen to efficiently coordinate ^89^Zr. After in vitro stability tests, we selected the chelator with longer arms, AAZTHAG, as the best complexing agent for ^89^Zr presenting a stability of 86.4 ± 5.5% in human serum (HS) for at least 72 h. Small animal PET/CT static scans acquired at different time points (up to 24 h) and ex vivo organ distribution studies were then carried out in healthy nude mice (*n* = 3) to investigate the stability and biodistribution in vivo of this new ^89^Zr-based complex. High stability in vivo, with low accumulation of free ^89^Zr in bones and kidneys, was measured. Furthermore, an activated ester functionalized version of AAZTHAG was synthesized to allow the conjugation with biomolecules such as antibodies. The bifunctional chelator was then conjugated to the human anti-HER2 monoclonal antibody Trastuzumab (Tz) as a proof of principle test of conjugation to biologically active molecules. The final ^89^Zr labeled compound was characterized via radio-HPLC and SDS-PAGE followed by autoradiography, and its stability in different solutions was assessed for at least 4 days.

## 1. Introduction

A rapidly expanding number of radionuclides with a variety of half-lives, emission types, and energies for the application of radionuclide imaging are routinely produced. When choosing the most suitable radionuclide for a certain application, one should not only consider the decay properties and availability of the radionuclide, but it is also of great importance that the physical half-life of the radionuclide matches the biological half-life of the vector molecule [1,2]. This biological half-life can be in the range of minutes (small organic molecules), hours (peptides, antibody fragments), or even days (full-size monoclonal antibodies). Longer-lived radioisotopes should be selected when an extended time is required to achieve optimal target-to-background ratios, expressed as the ratio between the accumulation of the radiotracer in the target tissue and the accumulation in muscle (as background organ). For diagnostic purposes, a radionuclide with relatively limited energy (100–200 KeV) and a high average path (typical γ rays) that can be detected by a detector near the patient is required. After the decay, the nuclide should lead to a low activity isotope that can be easily eliminated from the organism. Radioisotopes such as ^68^Ga, ^18^F, ^64^Cu, ^44^Sc, or ^89^Zr reflect these characteristics. One of the most important aspects of the development of metal-based PET probes is achieving a stable complexation of the radiometal to avoid its release in the blood pool, and to allow the delivery to a specific target in the body guaranteeing good diagnostic results. The study and development of new chelating ligands for commonly used radioisotopes is nowadays more focused on a series of cyclic, cross-bridged, and acyclic ligands that can lead to more stable and inert complexes with ^68^Ga, ^44^Sc, ^89^Zr, and ^64^Cu [3]. The well-known AAZTA chelator has already proven to be an interesting ligand for several metal ions due to the thermodynamically stable complexes formed with it [4,5]. In the past years, AAZTA-like derivatives have been synthesized for the successful complexation of ^68^Ga such as the recent PIDAZTA chelator and the CyAAZTA chelator [6,7]. Moreover, AAZTA was also used for preclinical PET application based on ^44^Sc radioisotope demonstrating the suitability of this chelating agent for the preparation of Sc-based radiopharmaceuticals [8,9]. Zirconium-89 (^89^Zr) is a second-row transition metal, and its potential application in PET imaging with ^89^Zr-based antibodies tracers was first demonstrated in 1992 [10]. Due to its long half-life (t_1/2_ = 78.4 h), the development of new radiotracers based on ^89^Zr has increased in recent years. In particular, zirconium-89 perfectly supports the development of radiotracers for immuno-PET that utilize immunoglobulin G (IgG) antibodies as targeting vectors, which require long periods (days to weeks) to fully accumulate at the target site in vivo [11]. Due to the fact that immunoPET has become the method of choice for imaging not only tumors but also immune cells, immune checkpoints, and inflammatory processes, the radiochemistry of Zr-89 and complexation strategies to use this radioisotope has driven the design and development of new chelators [12]. The main oxidation state of zirconium in aqueous solutions is +4 and for this reason, Zr(IV) (ionic radius = 0.84 Å) can be classified as a hard Lewis acid, and it is ideally complexed by hard Lewis bases, e.g., oxygen donor groups [13]. Since this tetravalent cation usually forms 6- and 8-coordinate complexes, nowadays the development of new ligands for ^89^Zr is based on the use of hexa and octadentate chelators with hydroxamate functional groups. The hydroxamate moiety is one of the best bidentate chelating groups forming a five-membered chelating ring around hard metal ions such as Zr^4+^ or Fe^3+^. From potentiometric studies, it was demonstrated that *N*-methylhydroxamate derivatives show improved coordination abilities to form stable Zr(IV)-complexes. Considering the similarities of Zr(IV) with Fe(III), the development of ^89^Zr chelators is commonly based on the natural complexes of Fe(III) [14]. Siderophores are high-affinity natural Fe(III)-chelating compounds designed to transport iron across cell membranes: they are amongst the strongest soluble Fe(III) binding agents known containing catecholates, hydroxamates, or (α-hydroxy-) carboxylates donor groups [15,16]. Thus, in the past years, new chelators for ^89^Zr have been developed containing hydroxamates, catecholates, and hydroxypyridinones coordinating groups [17]. Antibody radiolabeling with ^89^Zr is typically performed using the Desferrioxamine B (DFO) chelator, an FDA approved siderophore bearing three hydroxamate groups involved in the coordination of the metal [18]. However, the high flexibility of this linear chelator accounts for the inadequate stability of the ^89^Zr-DFO complex and has pushed the development of novel chelators based on macrocyclic structures able to form more inert complexes [19,20]. Moreover, since Zr(IV) can accommodate up to eight donors in its coordination sphere and DFO occupies only six coordination sites, octadentate non-macrocyclic chelators have also been designed and developed [21,22,23]. In particular, the octadentate version of the DFO chelator, the so-called DFO*, has been developed. Two different functionalized versions of this chelator were synthesized, conjugated with a monoclonal antibody, and tested in vivo showing a lower ^89^Zr bone uptake over the DFO conjugate [24]. Nowadays, the development of radiotracers for PET imaging is focused on the use of antibodies or antibodies fragments as carriers. Working with antibodies or their derivatives requests to take into account several issues: (i) antibodies are often pH-sensitive as well as heat-sensitive biomolecules; therefore, all reaction steps must be carried out in a reasonable pH range (4–9) and at controlled temperatures (e.g., 25, 37 °C), to prevent both the formation of irreversible c-structure tetramers and denaturing of the antibody; (ii) during the radiolabeling reactions, due to the sensitivity of antibodies to acidic pH values, it is necessary to work with neutralized solutions prior to radiolabeling [25]; (iii) the radiometal complex has to be stable enough over the time to achieve accumulation of the probe within the target tissue and allowing internalization after binding to the target antigen. In that case, a positron emitter is needed that residualizes in the target cell after internalization, like in the case of ^89^Zr, to enable imaging at optimal contrast. In this study, we synthesized and characterized two hexadentate AMPED-based ligands for ^89^Zr complexation investigating their in vitro and in vivo stability. A functionalized derivative of the most promising one was also synthesized and characterized to allow the conjugation with a humanized antibody and the subsequent ^89^Zr labeling. The stability of the radiolabeled immunoconjugate was then tested in vitro.

## 2. Results and Discussion

### 2.1. Ligand Synthesis

**Figure 1:**
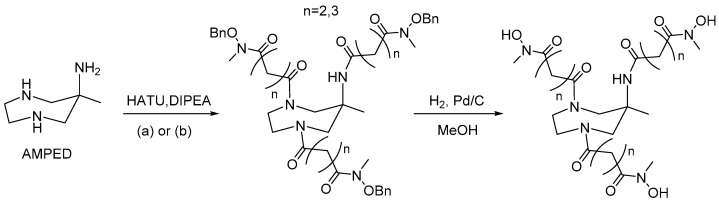
Brief synthesis scheme of AAZTHAS and AAZTHAG chelators. (**a**) 5-((benzyloxy)(methyl)amino)-2,5-dioxopentanoic acid (*n* = 2), (**b**) 6-((benzyloxy)(methyl)amino)-2,6-dioxohexanoic acid (*n* = 3).

In this work, we developed two hexadentate chelators for zirconium-89 starting from the heterocyclic structure of the triamine AMPED by insertion of three bidentate *N*-methylhydroxamate coordinating groups. The 6-amino-6-methyl-1,4-diazepine (AMPED) scaffold was synthesized using the established protocol via the double nitro-Mannich reaction between *N*,*N*-dibenzylethylenediamine, formaldehyde, and nitroethane, followed by simultaneous hydrogenation of the nitro group and hydrogenolysis of the benzyl moieties with H_2_ and Pd/C [4]. The pendant arm was synthesized following a reported procedure starting from *O*-benzyl-hydroxylamine [26]. Briefly, *O*-benzyl-hydroxylamine was protected with benzyl chloroformate, *N*-methylated with methyl iodide, deprotected at the nitrogen with hydrobromic acid (33% in AcOH), and finally acylated by succinic or glutaric anhydride to form the C4 or C5 carbon chains, respectively. The two different pendant arms were then coupled to the AMPED cycle by forming amide bonds using HATU (1-[Bis(dimethylamino)methylene]-1H-1,2,3-triazolo[4,5-b]pyridinium 3-oxide hexafluorophosphate) activator and DIPEA (*N*,*N*-diisopropylethylamine) to obtain *O*-benzyl protected AAZTHAS and AAZTHAG ligands as shown in Figure 1. AAZTHAS and AAZTHAG differ for the length of the spacers between the AMPED scaffold and the *N*-methylhydroxamate groups (succinic and glutaric moieties, respectively), based on previous studies where only a succinic spacer was used between the tetraazacyclotetradecane scaffold and the *N*-methylhydroxamate donors [20]. Furthermore, a recent paper by Klasen et al. reported a functionalized version of the chelator called AAZTHAS with the shorter spacer between the chelating units and the AMPED scaffold, showing a lower stability in human serum and PBS even when conjugated to a mAb [27]. As already mentioned in the introduction, the choice of a cyclic-based structure to hold the hydroxamate pendant arms relies on the higher rigidity given by this kind of scaffold to the final complex. In fact, it is reported in the literature that AMPED-based ligands are able to form stable complexes with various metal ions, being a good alternative for the development of tracers for PET imaging [8]. The final chelators were then obtained after hydrogenolysis of the *O*-benzyl groups. Labeling with ^89^Zr and stability tests in different solutions and human serum (Table 1) showed that ^89^Zr-AAZTHAG is more stable than ^89^Zr-AAZTHAS, therefore, the bifunctional AAZTHAG-C_5_-OH chelator bearing a tetrafluorophenol (TFP) activated ester to allow the conjugation with biomolecules via amide linkage was then synthesized. The higher stability of the tracer with longer arms highlights the quality of our design and confirms what was reported by Klasen et al. [27].

### 2.2. Prodution of AAZTHAG-C_5_-Tz

**Figure 2:**
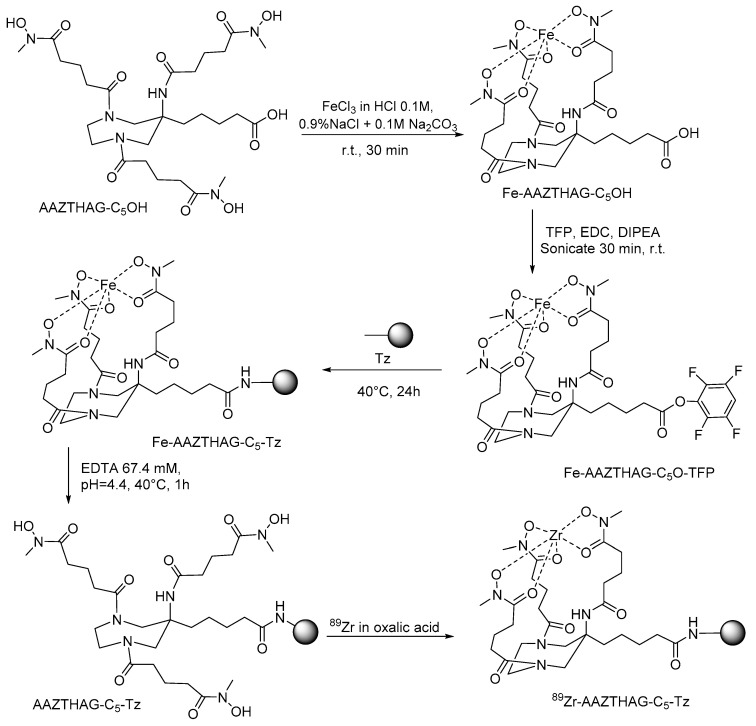
Synthesis scheme of ^89^Zr-AAZTHAG-C_5_-Tz.

Briefly, the AMPED scaffold bearing the three *N*-hydroxy-*N*-methylglutaramide pendant arms and an activated pentanoic acid group in 6-position of the cycle was synthesized with the aim to conjugate it to the biological carriers. The 6-position of the functional group is either due to easier synthetic access, as the cyclization was carried out using methyl 6-nitrohexanoate, or due to stereochemical and steric considerations, since the groups placed in this position should prevent any steric influence with the metal and retain the symmetry of the ligand avoiding the generation of stereocentres. Thus, the synthesis of the 6-methylpentanoate-AMPED derivative was performed as reported by Manzoni and colleagues, and then the amino groups were acylated by the *N*-(benzyloxy)-*N*-methylglutaric acid moieties using HATU activator and DIPEA as discussed earlier for the synthesis of AAZTHAG [28]. The final AAZTHAG-C_5_-OH ligand was obtained after LiOH mediated hydrolysis of the methyl ester followed by hydrogenolysis of the *O*-benzyl groups and semi-preparative HPLC-MS purification. The conjugation of the ligand to the –NH_2_ groups of Lys residues of the antibody requires the activation of the carboxylic acid group. We choose to activate it by forming a tetrafluorophenol ester using TFP and EDC (1-ethyl-3-(3-dimethylaminopropyl)carbodiimide), as TFP is more stable than *N*-hydroxysuccinimide group in basic conditions such as those used for the conjugation reaction to antibodies [29]. Briefly, as shown in Figure 2, it was first necessary to protect the hydroxamate groups of the pendant arms by complexing Fe(III) as already reported for the conjugation of mAbs with ^89^Zr via a tetrafluorophenol-N-succinyl-Fe-desferal ester [30]. Then, the activated ester of the complex, Fe-AAZTHAG-C_5_-OTFP, was formed by adding TFP/EDC as activators and conjugated to Trastuzumab (Herceptin^®^, Tz) in buffer NaHCO_3_ at pH = 9 at different temperatures (37–40 °C), times (30 min up to 24 h) and Ab concentrations (1.0 up to 8.0 mg/mL). The optimal conjugation conditions resulted in a conjugation reaction carried out at 40 °C, for 24 h and with a concentration of Ab of 8.0 mg/mL. These conditions differ from the protocol of Verel et al. since the reaction time is longer and the temperature slightly higher, in agreement with procedures reported by other authors [30]. In general, the yield of the conjugation step could be dependent on several parameters, such as the nature of the protein used, the incubation time, temperature, concentration of protein, concentration of chelator, and organic solvent used. Furthermore, the steric hindrance could play a role in this reaction: a semi-rigid chelator would most likely have a higher steric hindrance than the acyclic desferrioxamine B reported in the protocol from Verel and colleagues, reducing the reaction rate. Thus, in the present case, the reaction conditions were optimized to obtain a good recovery of the immunoconjugated product. Then, Fe(III) was removed from the tracer by transchelation reaction with EDTA at pH 4.4. We optimized this step avoiding the use of concentrated H_2_SO_4_ by carrying out the acidification with a buffer exchange using a solution of 0.25 M sodium acetate + gentisic acid 3 mM pH = 5.5 (called formulation buffer), and then adding EDTA at a concentration of 67.4 mM, as reported elsewhere [30]. The buffer exchange also allowed the purification of the product from the activated complex. The mixture was purified using PD-10 columns with formulation buffer that contains gentisic acid as a scavenger to avoid the radiolysis effect due to γ radiation, produced by ^89^Zr decay, and responsible for the oxidation of some protein sites, which might cause degradation of antibody structure with possible impairment of biological functions [31,32].

### 2.3. Determination of Chelator-to-Protein Ratio

The ratio between numbers of chelators AAZTHAG-C_5_-OH per molecule of Tz was measured indirectly via metal loading measurement carried out using ICP-MS. To this purpose, the cold ^nat^Zr-AAZTHAG-C_5_-Tz was synthesized by reaction of the final chelator-Tz conjugate with ^nat^Zr and purified on a PD-10 column. The complex was then subjected to both Bradford assay [A] and ICP-MS measurements [B] to determine protein and metal concentrations, respectively. Average values of 2.51 ± 0.04 × 10^−7^ M and 9.34 ± 0.03 × 10^−8^ M were obtained for [A] and [B], respectively. Then, the average number of chelators per Ab calculated as [A]/[B], resulted to be 2.6.

### 2.4. Radiolabeling and Characterization of AAZTHAG-C_5_-Tz

All labeling reactions were carried out following the procedure reported by Yusufi and colleagues, with slight modifications [33]. The products were then purified by a PD-10 column with formulation buffer as eluent and characterized with SEC-HPLC with γ and UV-VIS detectors. Since the concentration of the antibody can have a significant role during labeling reactions, we optimized the labeling protocol at different concentrations of the immunoconjugated compound. Thus, a radiolabeling yield (RCY) of 60 ± 15% with a specific activity of 7.1 ± 2.5 GBq/µmol was reached using a protein concentration of 8.0 mg/mL. The labeling yield was calculated using the formula:

$$\mathrm{RCY}\%=\frac{\mathrm{final}\;\mathrm{activity}\;\left(µ\mathrm{Ci}\right)}{\mathrm{initial}\;\mathrm{activity}\;\left(µ\mathrm{Ci}\right)}*100$$

**Figure 3:**
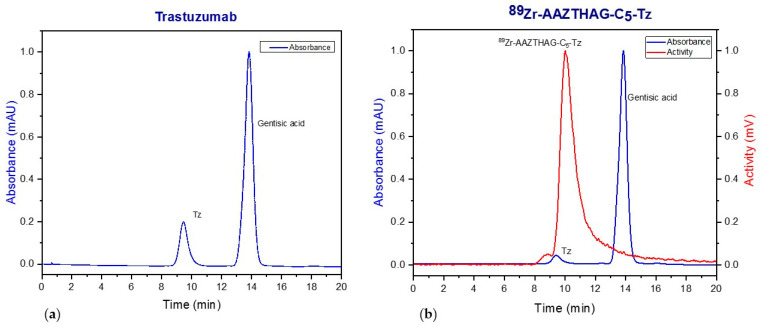
HPLC-SEC chromatogram of (**a**) Trastuzumab; (**b**) ^89^Zr-AAZTHAG-C_5_-Tz. UV–Vis: tr = 9.40 min; activity: tr = 10.00 min, the peak with tr = 13.38 min is due to Gentisic acid present in the formulation buffer used for the purification of the product. The radioactivity peak has the same retention time as the UV–Vis peak (the small delay between the two peaks is due to the sequential setup of the UV and radioactive detectors), proving that the antibody is conjugated to the ligand and that ^89^Zr is complexed by the ligand-antibody conjugate.

After conjugation, labeling and purification, the product ^89^Zr-AAZTHAG-C_5_-Tz was analyzed with SEC-radio-HPLC (Figure 3) and compared to Tz to show the successful conjugation and labeling of the tracer.

In addition, SDS-PAGE and autoradiography analysis confirmed the integrity of the radiotracer and the association of the radioactivity with the band correspondent to the Trastuzumab (Figure S3).

### 2.5. In Vitro Stability Studies

Stability studies were performed on ^89^Zr labeled AAZTHAS and AAZTHAG to test the effect of the pendant arm length on complex stability. The stability of the two ^89^Zr-complexes was investigated by radio-TLC after incubation for 72 h at 37 °C in formulation buffer, EDTA 5 mM (1000-fold excess), and human serum (HS). The percentage of ^89^Zr complexed measured in HS and in formulation buffer after 72 h for ^89^Zr-AAZTHAG was 87 ± 5%, and 73 ± 5%, respectively, indicating good in vitro stability of the compound. The stability was higher than that obtained for ^89^Zr-AAZTHAS equal to 46% in HS and 42% in the formulation buffer. These results suggested poor stability of the complex ^89^Zr-AAZTHAS showing a release of the radioisotope; furthermore, the complex tends to precipitate over time probably due to interaction with blood pool proteins. Based on these results, we carried on the study using the glutaramide derivative, since the longer length of the pendant arms resulted in a more stable ^89^Zr-complex.

In human serum, the amount of ^89^Zr associated with ^89^Zr-AAZTHAG after 3 days was 86.4 ± 5.5%. Based on these preliminary results, we also tested the stability of ^89^Zr-AAZTHAG in vivo (see Section 2.6). Then, the stability of the immunoconjugate derivative, ^89^Zr-AAZTHAG-C_5_-Tz, was also studied in vitro, showing a high percentage of Zr-89 retained by the complex for at least 4 days of incubation, both in formulation buffer and HS. These findings confirmed that even when functionalized and conjugated to a biomolecule (e.g., mAb) the chelator AAZTHAG can stably complex the Zr-89 isotope, even better than the correspondent non-functionalized version.

### 2.6. In Vivo Studies with ^89^Zr-AAZTHAG

**Figure 4:**
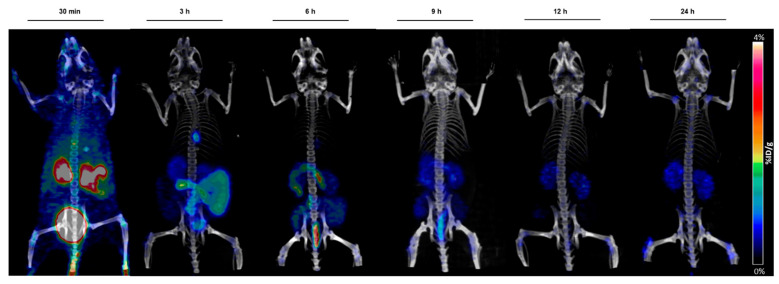
Maximum image projections (MIPs) obtained from static PET/CT scans at different time points. A hepatobiliary and renal excretion is clearly visible as well as a low accumulation in bones and kidneys 24 h p.i. Scale bar: 0–4%ID/g.

Based on the good preliminary in vitro stability results, we investigated the behavior of ^89^Zr-AAZTHAG in vivo in healthy female nude mice (*n* = 3) by acquiring PET/CT images at six different time points (30 min, 3 h, 6 h, 9 h, 12 h, and 24 h), and mice were then sacrificed 24 h p.i. to perform a biodistribution study. In Figure 4 below are reported the PET/CT images at the six time points, where a hepatobiliary and renal excretion is visible. Moreover, since a very low amount of Zr-89 has been released from the complex, a low signal in bones was visualized during the PET acquisition, indicating good stability in vivo, comparable to other compounds reported in the literature [21].

From a comparison between the biodistribution data of ^89^Zr-AAZTHAG and those described by Deri et al., on the HOPO chelator (Figure S4), it can be inferred that ^89^Zr-AAZTHAG presents higher stability and favorable low accumulation in selected organs such as liver, intestine, heart, muscle, bladder, and kidneys when compared to DFO, and a similar accumulation in femur and kidneys when compared to HOPO [21].

### 2.7. Ex Vivo Studies

**Figure 5:**
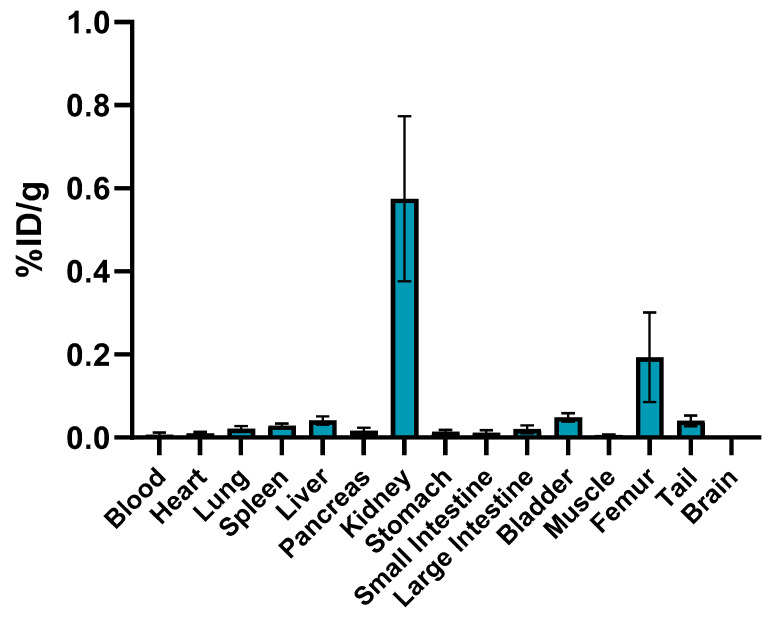
Biodistribution data of ^89^Zr-AAZTHAG on female healthy nude mice in selected organs measured 24 h p.i. The activity accumulated is reported as percentage of injected dose per gram (%ID/g ± SD) (*n* = 3).

Tracer accumulation results (Figure 5 and Figure S5) show high in vivo stability of the labeled complex ^89^Zr-AAZTHAG, as demonstrated by the low ^89^Zr accumulation measured in the femur (0.193 ± 0.108% ID/g), kidneys (0.575 ± 0.199% ID/g), and other organs at 24 h p.i. These results are comparable to those reported in the literature for in vivo stability studies of desferrioxamine (DFO) and another alternative chelator for Zr-89, the so-called HOPO chelator [21]. According to the PET images, our biodistribution data confirm the fast hepatobiliary/renal excretion of the labeled ligand due to the low molecular weight (558.63 Da) [34]. Although a “naked” and negatively charged ^89^Zr-chelate complex does not persist in vivo long enough to encounter a challenge to its structural integrity compared to a conjugated version, nevertheless, the results obtained show a stable ^89^Zr-complex once conjugated with a biomolecule. As already reported above, the functionalized version of this chelator was afterwards synthesized and conjugated to Tz, as a proof of concept. No damage occurred to the structure of the monoclonal antibody Trastuzumab once conjugated and radiolabeled with ^89^Zr, as confirmed by SDS-PAGE + autoradiography analysis and by stability analysis in vitro over time.

## 3. Materials and Methods

### 3.1. General

All chemicals were purchased from Sigma-Aldrich (Saint Louis, MI, USA) or Alfa Aesar (Heysham, UK) unless otherwise stated and were used without further purification. The ^1^H and ^13^C NMR spectra were recorded using a Bruker Advance III 500 MHz (11.4 T) spectrometer equipped with 5 mm PABBO probes and a BVT-3000 temperature control unit. Chemical shifts δ are reported relative to TMS and were referenced using the residual proton solvent resonances. HPLC analyses and mass spectra were performed on a Waters HPLC-MS system equipped with a Waters 1525 binary pump. Analytical measurements were carried out on a Waters XTerra MS C18 (5 μm 4.6 × 100 mm) and on a Waters C18 XTerra Prep (5 μm 19 × 50 mm) for preparative purposes. Electrospray ionization mass spectra (ESI MS) were recorded using an SQD 3100 Mass Detector (Waters), operating in positive or negative ion mode, with 1% *v*/*v* formic acid in methanol as the carrier solvent. The concentration of Trastuzumab and the immunoconjugated tracer (before labeling) were measured with an IMPLEN NanoPhotometer P330 (IMPLEN). For the purification of the final tracer before and after the labeling reaction, a PD-10 desalting column (Sephadex G-25 resin, GE Healthcare Life Sciences, London, UK) was used using a buffer solution of gentisic acid (2,5-dihydroxybenzoic acid) 3 mM + 0.25 M sodium acetate, pH = 5.5 (formulation buffer) as eluent. The immunoconjugated tracer (before and after labeling) was characterized by a Prominence HPLC system (Shimadzu, Kyoto, Japan) with a Photo Diode Array detector (Shimadzu) and a GABI Star γ detector (Raytest, Straubenhardt, Germany) with an SEC column Yarra 3 μm SEC-3000 (Phenomenex, Torrance, CA, USA) and isocratic elution with a PBS buffer (pH = 6.8) and a flow rate of 1 mL/min. The radiotracer was further characterized by SDS-PAGE in non-reducing conditions loading 20 µg of sample in each well, running the gel for 90 min at 100 V. The gel was stained using Coomassie Blue staining solution, followed by autoradiography measurement performed by exposing the gel to phosphorimaging plates (Fujifilm, Fuji, Tokyo, Japan) for 24 h. Read-out of the plate was performed with a Phosphor-imager (CR35 BIO, Dürr-Biomedical, Miami, FL, USA), and the radioactive signals associated with the bands corresponding to the intact tracer on the autoradiography images were analyzed using AIDA Image analyzer software. The ^89^Zr used during the labeling experiments was purchased from Perkin Elmer (Skovlunde, Denmark). The activity during experiments was measured with a Capintec CRC^®^ 15 R dose calibrator. Radiochemical yields (RCYs) were determined by radio-TLC using different elution solutions (Elution solution 1: 0.1 M sodium citrate pH = 5; Elution solution 2: ACN/H_2_O 7:3) and either TLC silica gel 60 plates (Merck Millipore, Burlington, MA, USA) or instant thin-layer chromatographic stripes (iTLC, Agilent). The stripes were read-out using a radio-TLC-scanner (Bioscan, Eckert and Ziegler, Brussels, Belgium) and data were analyzed by the Bio-Chrom Lite software. PET/CT scans of animals were performed using an Inveon Small Animal PET/CT scanner (Siemens, Knoxville, TN, USA). During ex vivo experiments, the activity accumulation in specific organs was measured using a γ-counter (Perkin Elmer).

### 3.2. Synthesis of AAZTHAS

The *N*-methyl-*N*-(benzyloxy)succinamide [26] protected arm (102 mg, 0.43 mmol, 4 eq) was dissolved in DMF (2 mL) and DIPEA (77 μL, 0.43 mmol, 4 eq) was added. The mixture was reacted for 5 min and then HATU (164 mg, 0.43 mmol, 4 eq) was added to activate the carboxylic acid. After 15 min, a solution of AMPED [4] (14 mg, 0.11 mmol, 1 eq) in MeOH was added dropwise and the mixture was reacted overnight at room temperature [20]. Then, AcOEt (+HCl 0.1 M) was added to the mixture and after 2 h the product was extracted in the organic phase, separated, dried, and evaporated under vacuum. The mixture was dissolved in MeOH to perform a hydrogenolysis reaction with Pd/C (10%) in an H_2_ atmosphere for 4 h under strong stirring. The mixture was then filtered on Celite^®^ 500 and washed well with MeOH. The final mixture was then purified with a preparative HPLC-MS with the following method: Flow: 20 mL/min H_2_O (+0.1% TFA)/MeOH; 0–3 min: from 5 to 40% B; 3–15 min: from 40 to 80% B; 15–16 min: from 80 to 100% B; 16–20 min: 100% B; 20–21 min from 100 to 5% B. The product (15 mg) was obtained in 27.3% yield. ^1^H NMR (Figure S6) (D_2_O, 500 MHz) δ(ppm): 1.29 (s, 3H, CH_3_-cycle), 2.52–2.72 (m, 3 × 4H, -CH_2_CH_2_CONOH), 3.40–3.86 (m, 4 × 2H, cycle), 3.13 (s, 9H, -CONCH_3_). ^13^C NMR (Figure S7) (D_2_O, 500 MHz) δ(ppm): 20.88 (CH_3_-cycle), 27.13–27.53 (-CH_2_CH_2_CONOH), 31.3–31.4 (-CH_2_CH_2_CONOH), 35.9 (-CONCH_3_), 48.94 (C-cycle), 46.43- 57.97 (cycle), 162.78–163.07 (-NHCOCH_2_-), 174.22- 174.71 (-CONOH). MS ESI^+^ m/z: 516.25 (C_21_H_36_N_6_O_9_ calculated), 517.53 [M + H]^+^ observed.

### 3.3. Synthesis of AAZTHAG

*N*-methyl-*N*-(benzyloxy)glutaramide [26] protected arm (106 mg, 0.43 mmol, 4 eq) was dissolved in DMF (2 mL) and DIPEA (77 μL, 0.43 mmol, 4 eq) was added. The mixture was reacted for 5 min and then HATU (164 mg, 0.43 mmol, 4 eq) was added to activate the carboxylic acid. After 15 min, a solution of AMPED [4] (14 mg, 0.11 mmol, 1 eq) in MeOH was added dropwise and the mixture was reacted overnight at room temperature [20]. Then AcOEt (+HCl 0.1 M) was added to the mixture and after 2 h the product was extracted in the organic phase, separated, dried, and evaporated under vacuum. The mixture was dissolved in MeOH to perform a hydrogenolysis reaction with Pd/C (10%) in an H_2_ atmosphere for 4 h under strong stirring. The mixture was then filtered on Celite^®^ 500 and washed well with MeOH. The final mixture was then purified with a preparative HPLC-MS with the following method: Flow: 20 mL/min H_2_O (+0.1% TFA)/MeOH; 0–3 min: from 5 to 40% B; 3–15 min: from 40 to 80% B; 15–16 min: from 80 to 100% B; 16–20 min: 100% B; 20–21 min from 100 to 5% B. The product (13 mg) was obtained in 20% yield. ^1^H NMR (Figure S8) (D_2_O, 500 MHz) δ(ppm): 1.41 (s, 3H, CH_3_-cycle), 1.88 (m, 3 × 2H -CH_2_C*H*_2_CH_2_CONOH), 2.45–2.56 (m, 3 × 4H, -C*H*_2_CH_2_C*H*_2_CONOH), 3.28 (s, 9H, -CONCH_3_), 3.60–4.20 (m, 4 × 2H, cycle). ^13^C NMR (Figure S9) (D_2_O, 500 MHz) δ(ppm): 20.27 (CH_3_-cycle), 20.85–21.55 (-CH_2_CH_2_CH_2_CONOH), 30.78–31.43 (-CH_2_CH_2_CH_2_CONOH), 32.11–32.41 (-CH_2_CH_2_CH_2_CONOH), 36.94 (-CONCH_3_), 49.15 (C-cycle), 46.29–58.30 (cycle), 162.90–163.20 (-NHCOCH_2_-), 174.98–175.42 (-CONOH). MS ESI^+^ *m*/*z*: 558.30 (C_24_H_42_N_6_O_9_ calculated), 559.64 [M + H]^+^ observed.

### 3.4. Synthesis of AAZTHAG-C_5_-Tz

#### 3.4.1. AAZTHAG-C_5_OH

The *N*-methyl-*N*-(benzyloxy)glutaramide [26] (88 mg, 0.35 mmol, 4 eq) was dissolved in DMF (1.8 mL) and DIPEA (60 μL, 0.35 mmol, 4 eq) was added. The mixture was reacted for 5 min and then HATU (133 mg, 0.35 mmol, 4 eq) was added to activate the carboxylic acid. After 15 min a solution of the functionalized AMPED [28] (20 mg, 0.087 mmol, 1 eq) in MeOH was added dropwise and the mixture was reacted overnight at room temperature [20]. Then AcOEt (+HCl 0.1 M) was added to the mixture and after 2 h the product was extracted in the organic phase, separated, dried, and evaporated under vacuum to obtain the intermediate compound I1 (Figure S2). The mixture was dissolved in MeOH adding a 5 mM solution of LiOH in MeOH/H_2_O and reacted overnight. The pH was adjusted to pH = 3 and the solvent was evaporated under reduced pressure. The product was washed with 10 mL of CH_2_Cl_2_ (×2), dried, filtered and the solvent was evaporated under reduced pressure to give 42 mg of the intermediate I2 (Figure S2). After hydrogenolysis reaction with Pd/C (10%) in an H_2_ atmosphere for 4 h under strong stirring, the final mixture was filtered on Celite^®^ 500 and then purified with a preparative HPLC-MS with the following method: Flow: 20 mL/min H_2_O (+0.1% TFA)/MeOH; 0–3 min: from 5 to 40% B; 3–15 min: from 40 to 80% B; 15–16 min: from 80 to 100% B; 16–20 min: 100% B; 20–21 min from 100 to 5% B. The product (6 mg) was obtained in 10% yield. ^1^H NMR (Figure S10) (D_2_O, 500 MHz) δ(ppm): 1.35 (d, 2H, -C_γ_H_2_-), 1.50 (m, 2H -C_δ_*H_2_*),1.59 (m, 2H, -C_β_*H_2_*-), 2.25 (m, 3 × 2H, -CH_2_CH_2_CH_2_CONOH), 2.41 (m, 2H, -C_α_*H_2_*COOH), 2.53 (m, 3 × 4H, -CH_2_CH_2_CH_2_CONOH), 3.25 (s, 3 × 3H, -CONCH_3_), 3.38–3.91 (m, 4 × 2H, cycle). ^13^C NMR (Figure S11) (D_2_O, 500 MHz) δ (ppm): 20.28 (-*C*_γ_H_2_*-*), 22.04 (-CH_2_*C*H_2_CH_2_CONOH), 24.31 (-*C*_β_H_2_*-*), 30.88 (-CH_2_*C*H_2_*C*H_2_CONOH), 31.39–32.4 (-*C*H_2_CH_2_CH_2_CONOH), 32.84–32.92 (-*C*_α_H_2_*-*), 33.4 (-*C*_δ_H_2_*-*), 35.93 (-CON*C*H_3_), 46.48–55.17 (AAZTA cycle), 174.9–175 (-*CO*CH_2_*C*H_2_*C*H_2_CONOH), 175.46–176.14 (-CH_2_*C*H_2_*C*H_2_*CO*NOH), 178.70 (-*CO*OH). MS ESI^+^ m/z: 644.34 (C_28_H_48_N_6_O_11_ calculated), 645.73 [M + H]^+^ observed.

#### 3.4.2. AAZTHAG-C_5_-OTFP

According to Verel and colleagues [35], 19 μL of a 41 mM solution of AAZTHAG-C_5_-OH were mixed with 170 µL of 0.9% NaCl (+0.1 M Na_2_CO_3_) and to this solution, 6.1 µL of FeCl_3_ 140 mM in 0.1% HCl was added to give a final concentration of AAZTHAG-C_5_-OH of 4 mM. After 30 min at room temperature under stirring the compound was freeze-dried. 0.78 μmol of the obtained Fe-AAZTHAG-C_5_-OH were dissolved in 170 μL of ACN/H_2_O and to this solution a 200 mg/mL ACN solution of TFP (17.1 μL, 20.6 μmol, 26.4 eq) and EDC (6.9 mg, 36.1 μmol, 43.3 eq) was added, pH = 5.8–6, carrying out to the formation of a brown-orange precipitate. The mixture was sonicated for 30 min at room temperature, and then the precipitate was washed with ACN/H_2_O and dried to obtain the Fe-AAZTHAG-C_5_-OTFP.

#### 3.4.3. AAZTHAG-C_5_-Tz

The chelator was then conjugated with Trastuzumab (Herceptin^®^) as follows. Briefly, a 21 mg/mL solution of Tz in PBS buffer (pH = 6.8) underwent a buffer exchange with a NaHCO_3_ buffer (pH = 9) using a 100 kDa Amicon Ultra 0.5 mL centrifugal filter unit (Merck Millipore). The concentration was then adjusted to 8 mg/mL and afterwards a 5 mM DMSO solution of Fe-AAZTHAG-C_5_-OTFP (5 eq) was added. The solution was kept in the dark and allowed to react under soft stirring for 24 h at 40 °C. The final solution underwent an additional buffer exchange step with an Amicon Ultra centrifugal filter (Merck) and formulation buffer (pH = 4.4) and then a 67.4 mM solution of EDTA (50 eq) was added. The demetallation reaction was carried out at 40 °C for 1 h. The product was then purified using a PD-10 column and formulation buffer (pH = 5.5). The antibody recovery after conjugation (up to 80%) was calculated comparing the initial and final concentration of the carrier species measured with the Nanophotometer as reported below:

$$\mathrm{Ab}\;\mathrm{recovery}\;\left(\%\right)=\frac{{\left[\mathrm{Ab}\right]}_{f}}{{\left[\mathrm{Ab}\right]}_{i}}*100$$

### 3.5. Radiolabeling Experiments

AAZTHAS, AAZTHAG, and AAZTHAG-C_5_-Tz were labeled with ^89^Zr (Perkin Elmer) as reported elsewhere [33] and radiochemical purity (RCY) after PD-10 purification assessed by radio-HPLC and by radio-TLC.

#### 3.5.1. Synthesis of ^89^Zr-AAZTHAS and ^89^Zr-AAZTHAG

The labeling reactions were performed with a ratio of 80 μg of the compound to 1 mCi ^89^Zr in 100 μL oxalic acid 1 M (eventually adjusting the volume with oxalic acid 1 M). The ^89^Zr-oxalate solution was first neutralized with 45 μL of Na_2_CO_3_ 2M and incubated for 3 min at room temperature, and then 155 μL of HEPES (pH = 7.0) was added. Either AAZTHAS or AAZTHAG was added to the solution and the pH was adjusted with 350 μL of HEPES buffer to pH = 7.0. The solution was incubated at 37 °C for 30 min and RCYs were afterwards calculated by radio-TLC (Elution solution 2). The products were purified using a Sep-Pak Alumina N Plus Light cartridges preconditioned with 5 mL of 0.9% NaCl solution and then eluted with 1.5 mL of 0.9% NaCl solution.

#### 3.5.2. Synthesis of ^89^Zr-AAZTHAG-C_5_-Tz

Three different concentrations of the immunoconjugated product (1.0, 2.0, and 5.0 mg/mL) were tested. The labeling reactions were performed with a ratio of 500 μg of AAZTHAG-C_5_-Tz to 1 mCi ^89^Zr in 100 μL oxalic acid 1 M (eventually adjusting the volume with oxalic acid 1 M). The ^89^Zr-oxalate solution was first neutralized with 45 μL of Na_2_CO_3_ 2 M and incubated for 3 min at room temperature, and then 155 μL of HEPES (pH = 7.0) was added. At this point, the AAZTHAG-C_5_-Tz was added to the solution and the pH was adjusted with 350 μL of HEPES buffer to pH = 7.0. The solution was incubated at 37 °C for 30 min and the product was purified using a PD-10 desalting column with formulation buffer, collecting the eluate in different fractions of about 700 μL (20 drops). RCY was calculated by radio-TLC (Elution solution 1). The product was then characterized by SEC-HPLC with isocratic elution using PBS buffer (pH = 6.8) as solvent.

### 3.6. In Vitro Stability Studies

After the purification step and analysis of the radioactive complex via radio-HPLC, the stability assessment of the complex at different time points and in different solutions was carried out. ^89^Zr-AAZTHAS and ^89^Zr-AAZTHAG were dissolved in formulation buffer, EDTA 5 mM, and human serum in 1:5 *v*/*v* ratio and were incubated at 37 °C up to 96 h. At each time point the stability was evaluated by radio-TLC (Elution solution 2) using TLC silica gel 60 plates (Merck Millipore).^89^Zr-AAZTHAG-C_5_-Tz was dissolved in formulation buffer, EDTA 5 mM and human serum in 1:5 *v/v* ratio and was incubated at 37 °C up to 96 h. At each time point the stability was evaluated by radio-iTLC (Elution solution 1) using an instant thin-layer chromatographic strip (iTLC, Agilent).

### 3.7. Determination of Chelator-to-Protein Ratio

The metal loading of the conjugate was determined by complexing 0.5 mg of AAZTHAG-C_5_-OH with ^Nat^Zr(IV) (ZrCl_4_, 162 µg, 0.9 eq), for 30 min at room temperature. After lyophilization, ^Nat^Zr-AAZTHAG-C_5_-OH was dissolved in 170 μL of ACN/H_2_O and to this solution, 17.1 μL of a 200 mg/mL ACN solution of TFP (20.6 μmol, 26.4 eq) and 6.9 mg of EDC (36.1 μmol, 43.3 eq) were added (pH = 5.8–6.0) and carried out until the formation of a brown-orange precipitate. The mixture was sonicated for 30 min at room temperature, and the precipitate was washed with ACN/H_2_O and dried to obtain the ^Nat^Zr-AAZTHAG-C_5_-OTFP. The product was then conjugated with Trastuzumab. To a NaHCO_3_ buffer solution of Tz (pH = 9.0) a 5 mM DMSO solution of ^Nat^Zr-AAZTHAG-C_5_-OTFP (5 eq) was added and was reacted at 40 °C for 24 h under soft stirring and kept in the dark. The conjugate was then purified using a PD-10 column and formulation buffer (pH = 5.5) and the collected fractions were then subjected to both a Bradford assay [A] to determine the protein concentration and to ICP-MS measurements [B] to determine the metal concentrations. Regarding ICP-MS experiments, the fractions were digested with HNO_3_ (69% *w*/*w*) for 3 h at 65 °C in an ultrasonic bath. After the completion of the mineralization run and the cooling to room temperature, the content was transferred into a marked flask using HNO_3_ 1%. Metal quantification was measured by inductively coupled plasma-mass spectrometry (ICP-MS, Thermo Optek X Series 2).

### 3.8. Animal Studies

#### 3.8.1. In Vivo Studies with ^89^Zr-AAZTHAG

In order to study the biodistribution and the in vivo stability of the ^89^Zr-AAZTHAG, 8 weeks old pathogen-free female athymic Nude-Foxn1nu/nu mice (Charles River Laboratories, Sulzfeld, Germany) (*n* = 3) were injected via a catheter in the tail vein with 2.9 ± 0.1 MBq of ^89^Zr-AAZTHAG in 300 μL NaCl 0.9% solution, and imaged at different time points (30 min, 3 h, 6 h, 9 h, 12 h, 24 h) via PET/CT static acquisition using an Inveon Small Animal PET/CT scanner (Siemens, Knoxville, TN, USA).

#### 3.8.2. Ex Vivo Tracer Accumulation Analysis

Biodistribution studies were also performed to assess the in vivo distribution of the ^89^Zr-AAZTHAG complex and evaluate its in vivo stability. After sacrifice, the mice by anesthesia, blood, and other selected organs were collected, weighed, and the activity was measured by a γ-counter. The tracer accumulation in selected organs was expressed as a percentage of injected dose per gram of tissue (%ID/g).

## 4. Conclusions

New pseudo-macrocyclic ligands for ^89^Zr complexation for PET imaging were synthesized, in particular, two non-functionalized chelators, AAZTHAS and AAZTHAG, and one functionalized for conjugation to biomolecules, AAZTHAG-C_5_-OTFP. All three chelators are based on N-methylhydroxamate coordination groups accordingly to the coordination sphere of Zr^4+^. Preliminary labeling and in vitro stability studies were performed, focused on testing the effect of the length of the spacer arms in the AAZHTAS and AAZTHAG chelators. The initial hypothesis was that longer pendant arms would better coordinate the Zr-89 isotope, leading to a more stable complex over time. In fact, while the complex ^89^Zr-AAZTHAS precipitates when incubated in HS, the chelator AAZTHAG formed an ^89^Zr-complex stable in HS for at least 4 h. Very recent work from Klasen and colleagues, which showed low stability both in PBS ad human serum of the functionalized version of AAZTHAS conjugated to a mAb, supports our results [27]. A functionalized version of the AAZTHAG chelator was then synthesized since it was confirmed that a longer spacer arm leads to a more stable ^89^Zr-complex. The protocol used for the conjugation reaction between the ligand and the antibody was optimized with the aim to increase both reaction and labeling yield, avoiding the biomolecules to be subjected to strong reaction conditions. The optimized procedure allowed to obtain a final RCY of 41% after the labeling reaction of AAZTHAG-C_5_-Tz. Based on the obtained results, future work can be directed towards two main lines: (1) focusing on a direct conjugation approach of the chelator to the biomolecule avoiding the “protection/deprotection” steps consisting of the Fe(III) complexation and EDTA transchelation reactions. This can be obtained, for example, by modifying the functional group from a carboxylic acid to isothiocyanate or maleimide groups. In fact, the introduction of these functional groups could reduce the time of the conjugation/labeling procedure being a valid alternative for the conjugation reaction with mAb [1,18,36]. (2) Another modification that could be applied to the ligand is the introduction of a fourth pendant arm leading to an octadentate chelator that could be, together with the use of cyclic chelators, the key to produce a highly stable complex as recently reported for the DFO derivative DFO* [2,37] For this purpose, a new synthetic strategy must be planned.

## Figures and Tables

**Table 1:** In vitro stability analysis of ^89^Zr-AAZTHAS, ^89^Zr-AAZTHAG and ^89^Zr-AAZTHAG-C_5_-Tz. All the complexes were incubated at 37 °C in formulation buffer (3 mM gentisic acid + 0.25 M NaOAc, pH = 5.5), human serum and EDTA 50 mM solution. Two µL of each radiolabeled complex were spotted onto: (a) a thin-layer chromatography strip (TLC) eluted using elution solution 2; ^89^Zr-AAZTHAS/^89^Zr-AAZTHAG migrate along the TLC strip (Rf = 0.9), while free ^89^Zr remains at the origin (Rf = 0.0); (b) an instant thin-layer chromatography strip (iTLC) eluted using elution solution 1; free ^89^Zr migrates along the iTLC strip (Rf = 0.9), while ^89^Zr-AAZTHAG-C_5_-Tz remains at the origin (Rf = 0.0). Data are expressed as % of ^89^Zr complexed over total activity measured (^89^Zr complexed + free ^89^Zr).

^89^Zr Tracer	Incubation Solution	Day 0	Day 1	Day 2	Day 3	Day 4
AAZTHAS	Formulation buffer HS EDTA	71.0 71.0 15.1	63.3 76.8 1.0	62.3 59.2 1.6	41.4 1.5	n.a. ^1^ n.a. n.a.
AAZTHAG	Formulation buffer HS EDTA	91.5 ± 10.6 92.0 ± 11.3 30.6 ± 30.3	83.0 ± 6.5 77.7 ± 18.6 4.4 ± 3.9	78.1 ± 13.8 76.9 ± 15.4 2.4 ± 0.1	73.0 ± 5.2 86.4 ± 5.5 3.2 ± 2.6	n.a. n.a. n.a.
AAZTHAG-C_5_-Tz	Formulation buffer HS EDTA	n.a. n.a. n.a.	100.0 99.5 22.3	99.6 98.0 20.0	99.5 96.0 18.6	98.8 95.4 8

^1^ Not applicable.

## Data Availability

The data are available on request from the corresponding author.

## Supplementary Materials

The following are available online. Figure S1: Synthesis scheme of AAZTHAS and AAZTHAG, Figure S2: Synthesis scheme of AAZTHAG-C_5_-OH, Figure S3: SDS-PAGE and autoradiography of ^89^Zr-AAZTHAG-C_5_-Tz, Figure S4: Comparison between biodistribution data of ^89^Zr-AAZTHAG with ^89^Zr-DFO and ^89^Zr-HOPO, Table S5: Biodistribution data of ^89^Zr-AAZTHAG, Figures S6–S11: ^1^H and ^13^C NMR spectra of final ligands.

## Sample Availability

Samples of the compounds are not available from the authors.
