# Supplementary material for: A Semi Rigid Novel Hydroxamate AMPED-Based Ligand for 89Zr PET Imaging
Source: Molecules. 2021 Sep 25;26(19):5819. doi: 10.3390/molecules26195819 (PMC8512011; doi:10.3390/molecules26195819)
Supplement: Supplementary file 1 [file molecules-26-05819-s001.zip › molecules-1341443-supplementary.pdf]

molecules

MDPI

Supplementary Materials

A semi rigid novel hydroxamate AMPED-based ligand for ^{89}*Zr PET imaging*

Lisa Russelli [1], Francesco De Rose [1], Loredana Leone [2], Sybille Reder [1], Markus Schwaiger [1], Calogero D'Alessandria [1,*#] and Lorenzo Tei [2,*#]

[1] Department of Nuclear Medicine, Klinikum rechts der Isar TU München, Ismaningerstraße 22, 81675, Munich, Germany; calogero.dalessandria@tum.de

[2] Department of Science and Technological Innovation, Università del Piemonte Orientale, Viale T. Michel 11, 15121, Alessandria, Italy; lorenzo.tei@uniupo.it

* Correspondence: L.T. lorenzo.tei@uniupo.it; C.D. calogero.dalessandria@tum.de

[#] Join senior authors

Supplementary Materials: The following are available online at www.mdpi.com/xxx/s1, Figure S1: Synthesis scheme of AAZTHAS and AAZTHAG, Figure S2: Synthesis scheme of AAZTHAG-C5-OH, Figure S3: SDS-PAGE and autoradiography of ^{89}Zr-AAZTHAG-C5-Tz, Figure S4: Comparison between biodistribution data of ^{89}Zr-AAZTHAG with ^{89}Zr-DFO and ^{89}Zr-HOPO, Table S5: Biodistribution data of ^{89}Zr-AAZTHAG, Figure S6-11: ^{1}H and ^{13}C NMR spectra.

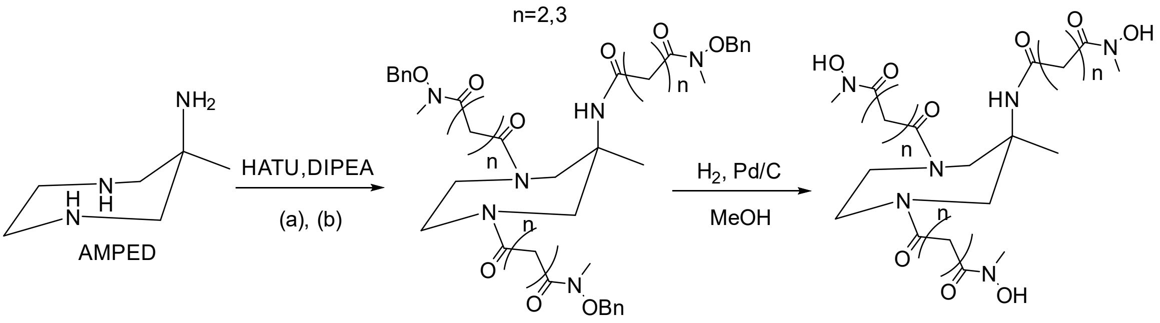

Figure S1: Synthesis scheme of AAZTHAS (n=2) and AAZTHAG (n=3): (a): *N*-methyl-*N*-(benzyloxy)succinamide protected arm, (b): *N*-methyl-*N*-(benzyloxy)glutaramide protected arm

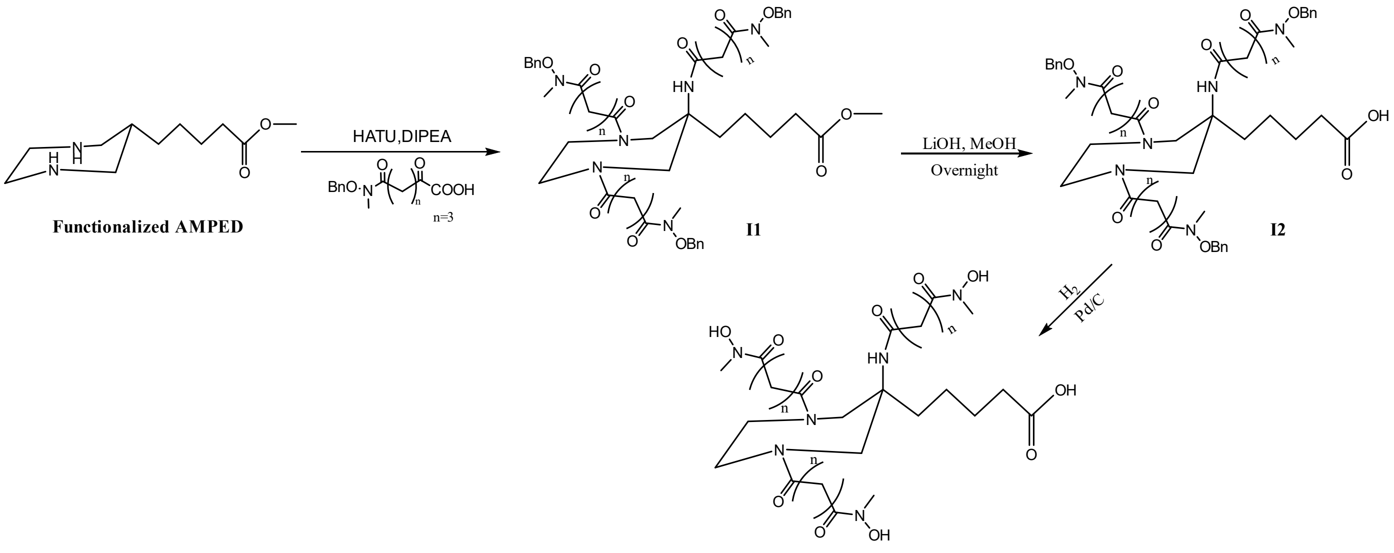

AAZTHAG-C$_5$OH

Figure S2: Synthesis scheme of AAZTHAG-C_5-OH

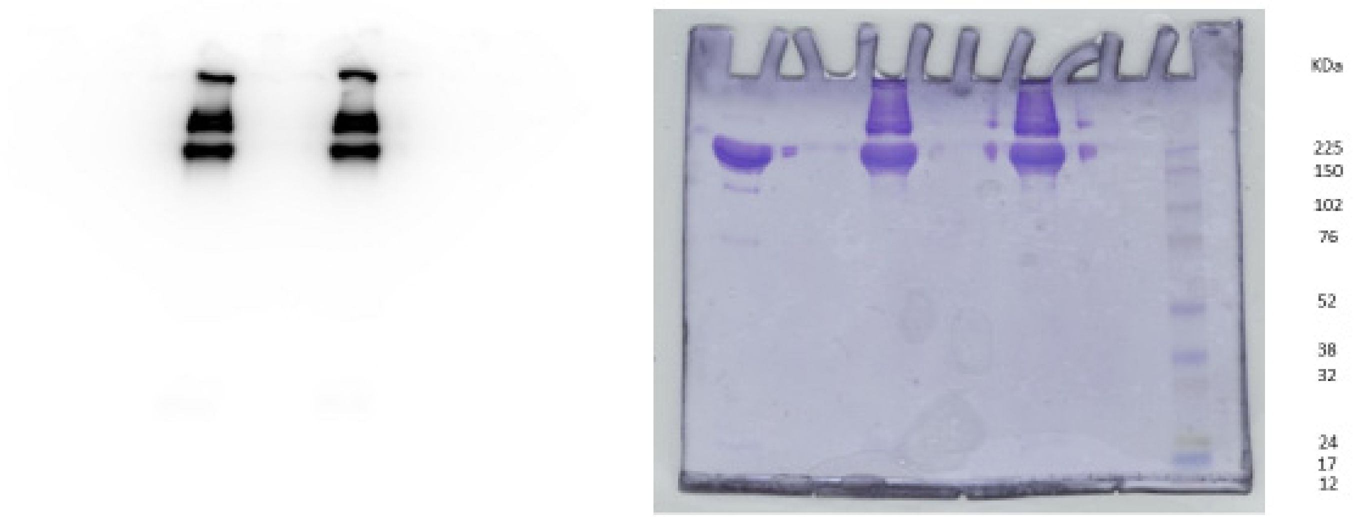

Figure S3: SDS-PAGE and relative Autoradiography of *^{89}Zr-AAZTHAG-C_5-Tz*. First band on the left is associated to Trastuzumab as reference. Two different bands are visualized on the gel corresponding to formation of two species of the radiolabelled Trastuzumab with different molecular weight: the monomeric one (150 kDa) and the dimeric one (250 kDa). The radioactivity is associated to both fractions.

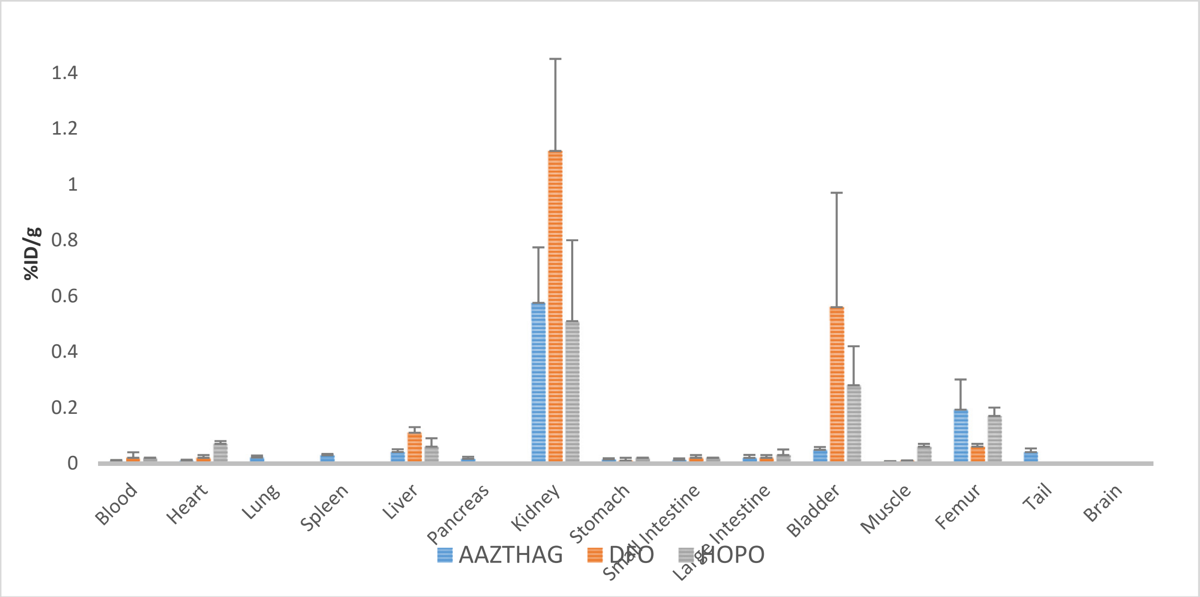

Figure S4: Comparison between biodistribution data of ^{89}Zr-AAZTHAG with ^{89}Zr-DFO and ^{89}Zr-HOPO.

**Organ**	**%ID/g**
Blood	0.007 ± 0.005
Heart	0.010 ± 0.004
Lung	0.021 ± 0.007
Spleen	0.029 ± 0.005
Liver	0.041 ± 0.010
Pancreas	0.016 ± 0.008
Kidney	0.575 ± 0.199
Stomach	0.014 ± 0.005
Small Intestine	0.012 ± 0.006
Large Intestine	0.020 ± 0.010
Bladder	0.049 ± 0.010
Muscle	0.006 ± 0.002
Femur	0.193 ± 0.108
Tail	0.040 ± 0.013
Brain	0.002 ± 0.001

Table S5: Biodistribution data of ^{89}Zr-AAZTHAG on female healthy nude mice in selected organs measured 24 h p.i.. The radioactivity accumulated in organs is reported as Percentage of Injected Dose per gram (%ID/g±SD) (n=3).

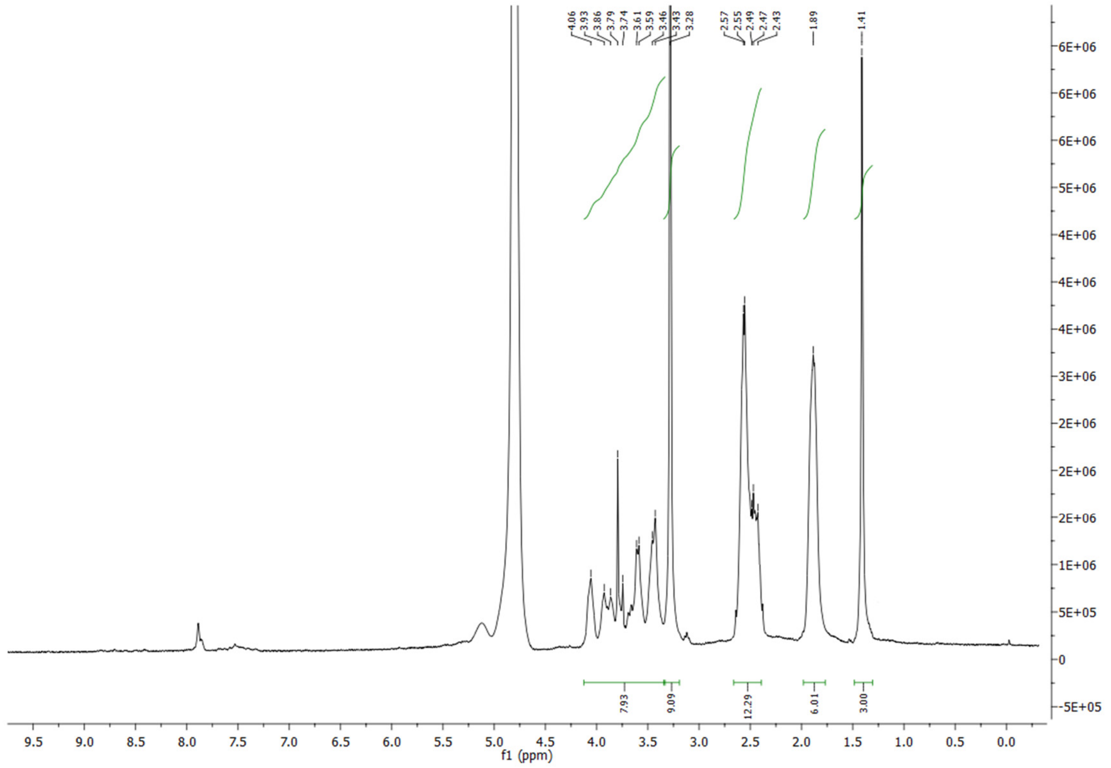

Figure S6: ^{1}H NMR spectrum of AAZTHAS

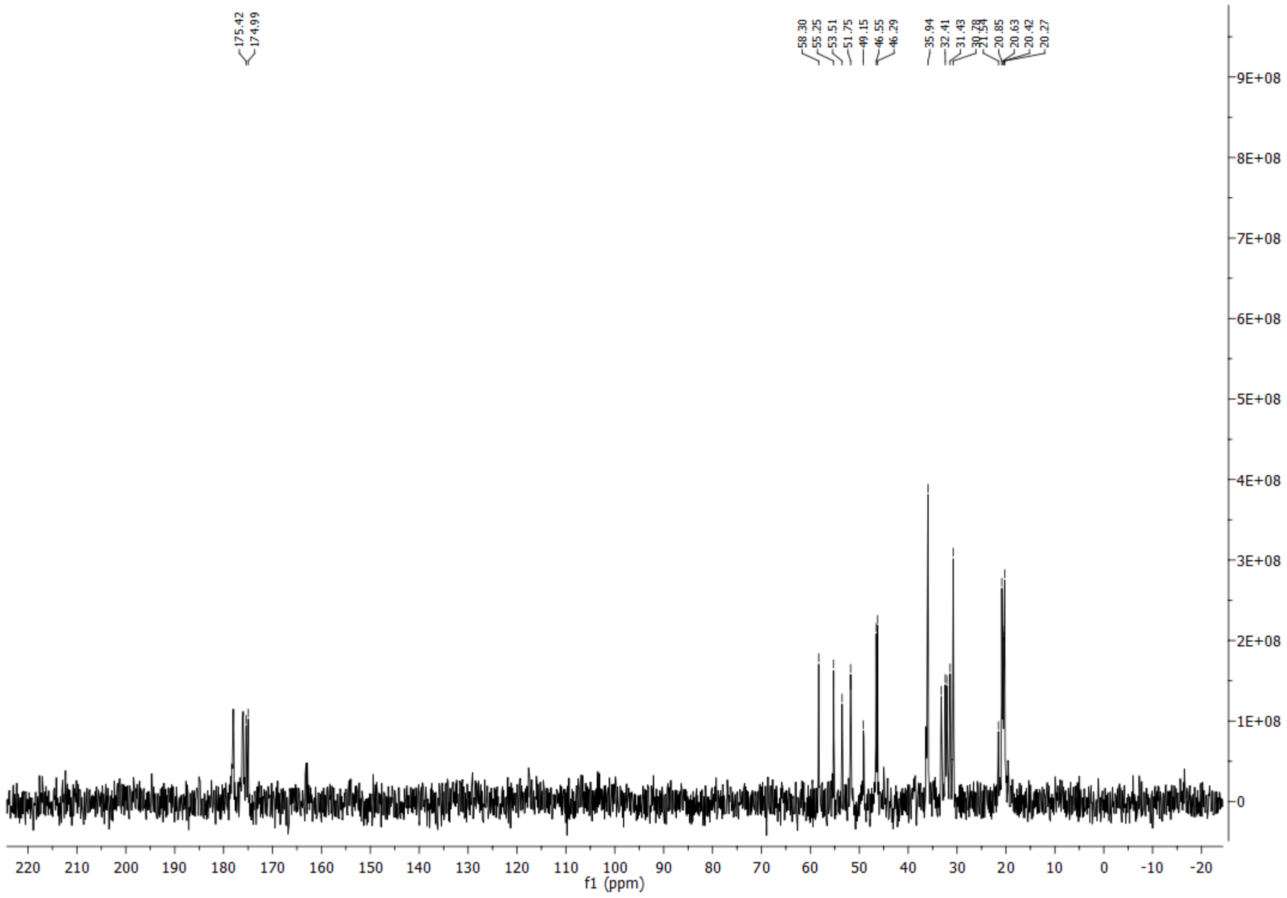

Figure S7: ^{13}C NMR spectrum of AAZTHAS

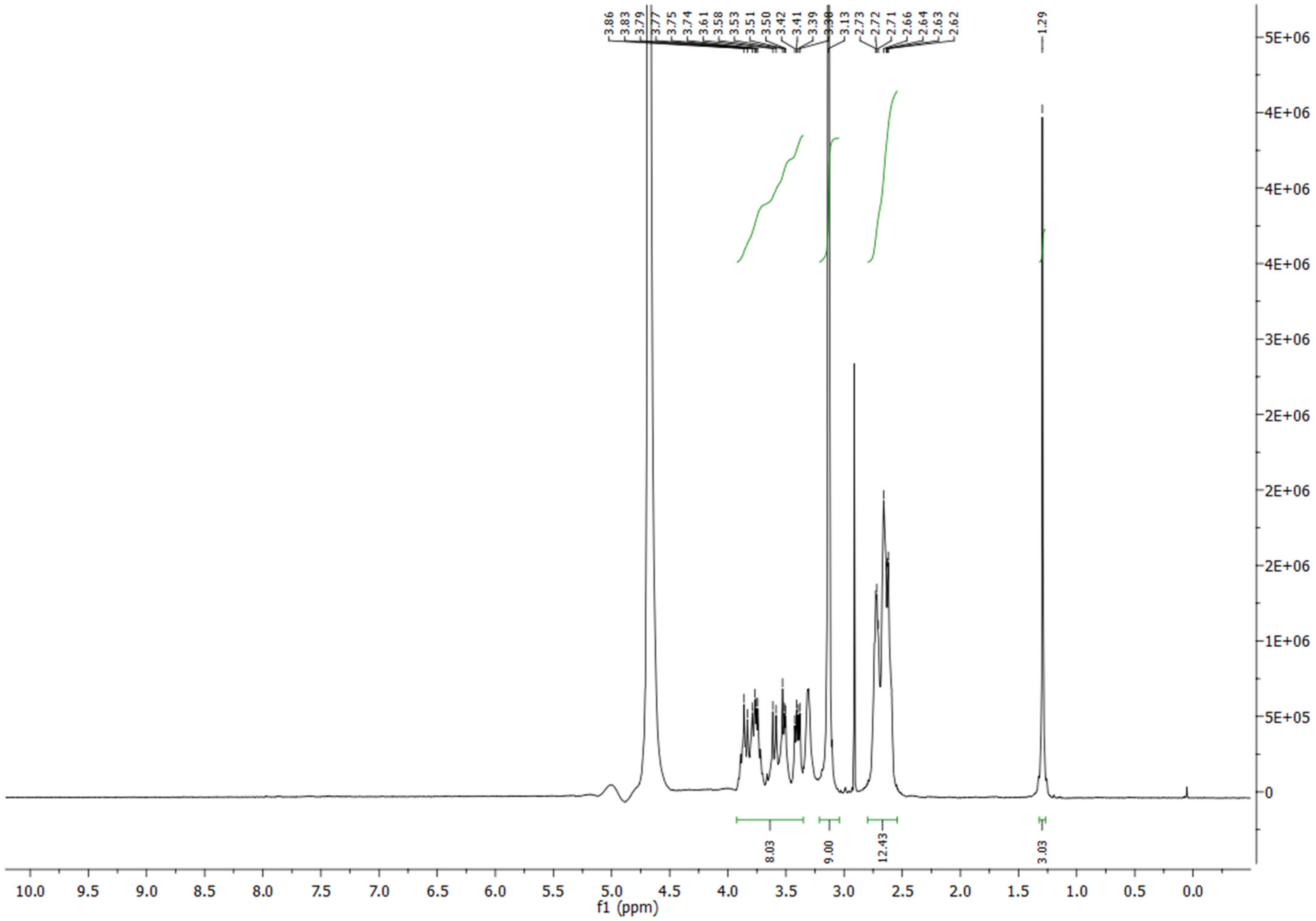

Figure S8: ^{1}H NMR spectrum of AAZTHAG

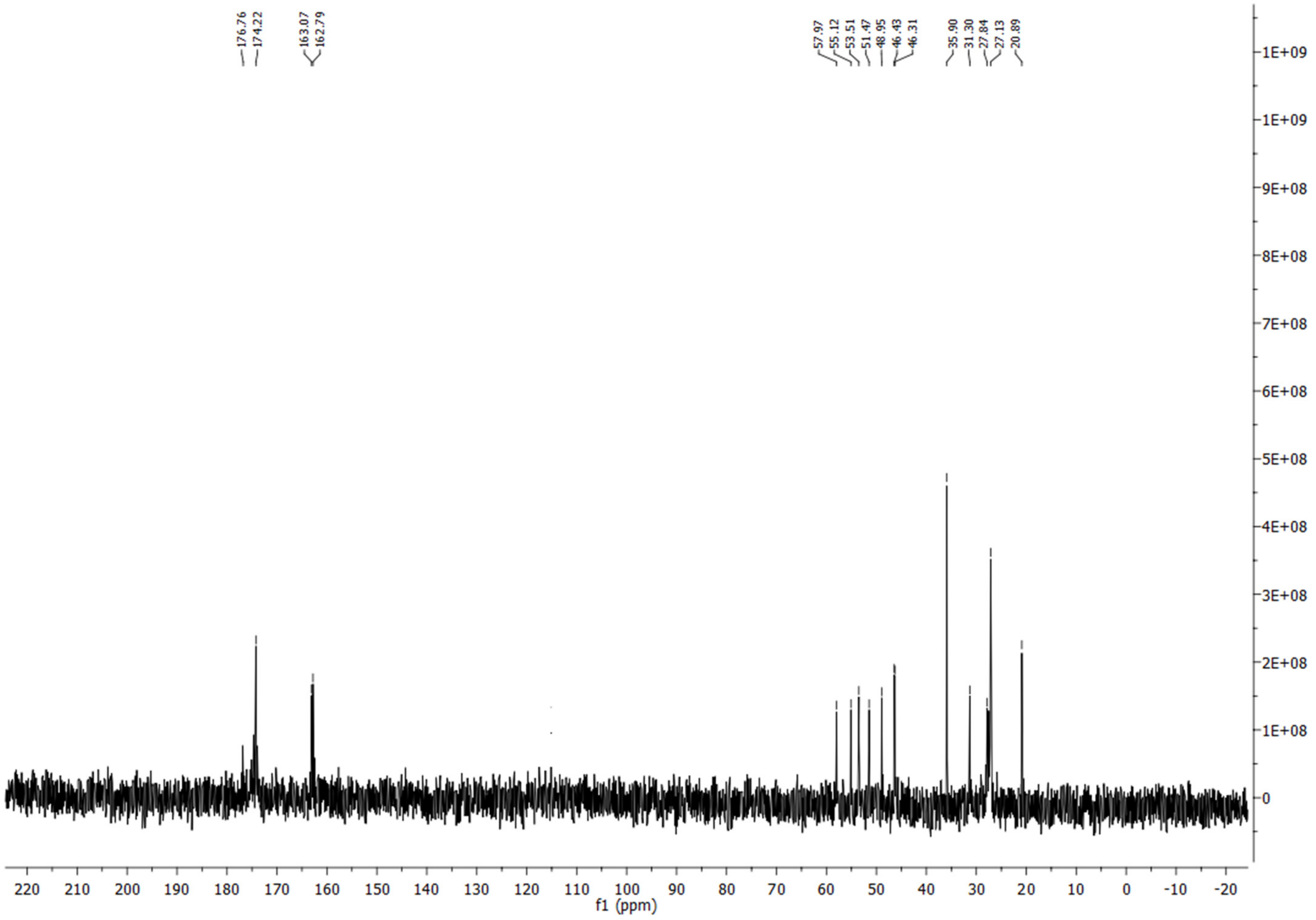

Figure S9: ^{13}C NMR spectrum of AAZTHAG

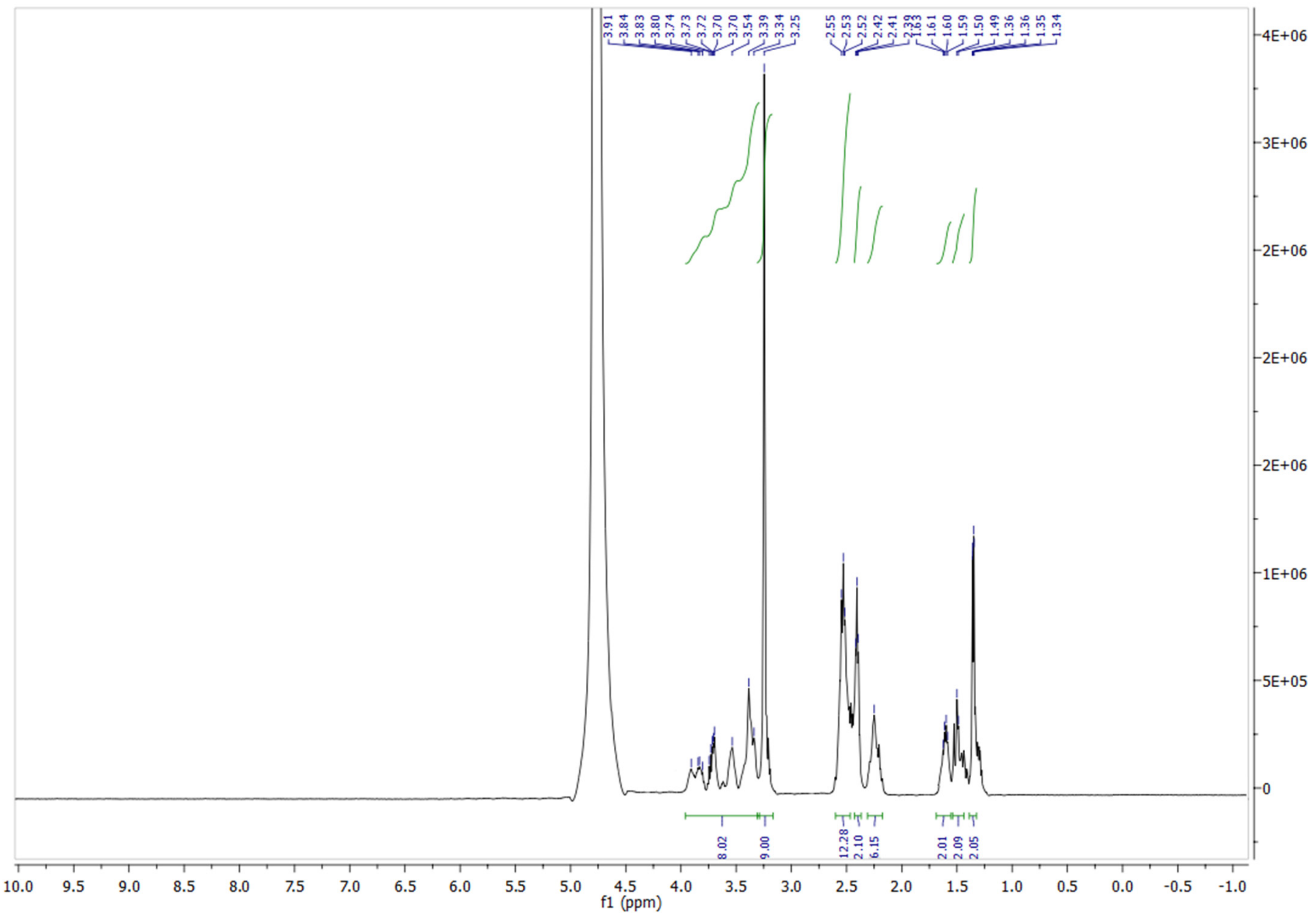

Figure S10: ^{1}H NMR spectrum of AAZTHAG-C_5OH

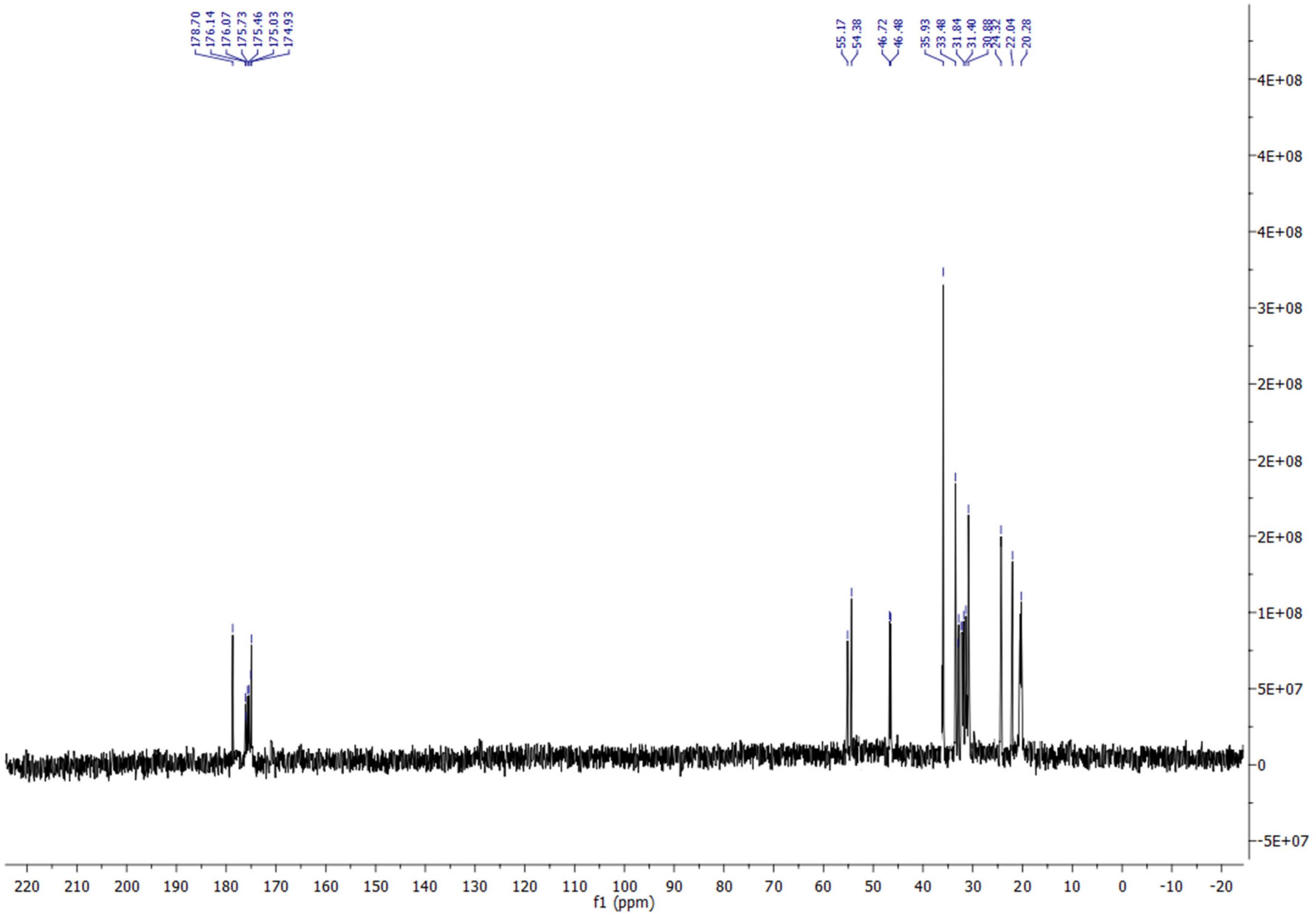

Figure S11: ^{13}C NMR spectrum of AAZTHAG-C_5OH
